# Editorial: Contextual-behavioral approaches to improving well-being and mental health in chronic physical illness

**DOI:** 10.3389/fpsyg.2024.1497106

**Published:** 2024-10-25

**Authors:** Sérgio A. Carvalho, Marcela Matos, Nuno Ferreira, Christopher D. Graham

**Affiliations:** ^1^University of Coimbra, Center for Research in Neuropsychology and Cognitive-Behavioral Interventions, Coimbra, Portugal; ^2^School of Humanities and Social Sciences, University of Nicosia, Nicosia, Cyprus; ^3^Department of Psychological Sciences & Health, University of Strathclyde, Glasgow, United Kingdom

**Keywords:** contextual-behavioral science, chronic physical illness, Acceptance and Commitment Therapy, self-compassion, meta-analysis, randomized controlled trial, mixed-method designs

Contextual Behavioral Science (CBS) is a principle-focused multi-level approach to the science of the human condition, and its practical application, which integrates contextual philosophy, evolution-informed research, and behavioral analytic principles (Hayes et al., 2012a). The primary therapeutic model to emerge from CBS is arguably Acceptance and Commitment Therapy (ACT; Hayes et al., 2012b), which promotes a quality within behavior called psychological flexibility. ACT engenders psychological flexibility through the use of techniques that promote awareness, willingness and valued action.

There have been at least 45 trials of ACT in chronic physical illnesses (CPI) like cancer, neurological diseases, and cardiovascular disease, with results supporting efficacy for improving quality of life and symptom burden (Konstantinou et al., 2023). In chronic pain conditions, there have been many trials of ACT, with results supporting efficacy for improving functioning and symptom interference (Lai et al., 2023), such that ACT is now listed as an “empirically supported treatment” for chronic pain by the American Psychiatric Association.

## What is next for CBS research in CPI?

More evidence is needed on the efficacy of a broader range of CBS therapies and how they can be integrated into a process-based approach to CPI (e.g., Ong et al., 2024). Also, little is known about how PF responds to therapeutic intervention and manifests in participant behavior to influence outcomes, due to the need for improved measures of PF that do not conflate this process with traditional outcomes (e.g., distress; quality of life) (Doorley et al., 2020). Furthermore, there is a need to link therapeutic techniques to changes in participant behavior and evaluate change at the individual level, as opposed to traditional trial methods that tend to prioritize the measurement of group or average change (Hayes et al., 2023). Also, the multi-level multi-factorial approach to the human condition followed by CBS requires further investment in the exploration of biological parameters of health and illness when conducting efficacy studies (Gloster et al., 2020).

This Research Topic examines the science of CBS approaches applied to CPI, aims to add new evidence on the efficacy of CBS interventions in CPI to the literature and aims to contribute to bridging the gap between several levels of the CBS approach by welcoming studies with qualitative methodologies, biological data analyses, and integrative CBS approaches to CPI.

## Expanding CBS in CPI: multidisciplinary and integrative approach

Poli et al. conducted an efficacy study of a CBS approach (ESPRIMO) with Italian young adults with multiple sclerosis (MS) to improve participants′ quality of life. The development of their intervention followed a co-creative process by engaging stakeholders in an online survey, a focus group and an advisory board. Implementation of ESPRIMO was feasible, and results showed a significant 5-fold increase in quality of life and functioning from pre- to post-intervention.

## The CBS approach to irritable bowel disease: different formats and biological outcomes

Ferreira, Pereira, Skvarc et al. tested the efficacy of LIFEwithIBD, in an RCT design in Portuguese individuals with IBD. Both conditions (LIFEwithIBD and waiting list control) improved stress and IBD symptom perception, but LIFEwithIBD participants presented significantly lower Crohn's disease Symptom severity at follow-up (3 months). In a commendable effort at a multi-level biopsychosocial approach, Ferreira, Pereira, Skvarc et al. tested the impact on several biomarkers. The results found no significant difference, although minor non-clinically significant changes were found in albumin at 1-year follow-up. Ferreira, Pereira, Matos-Pina et al. also reported an RCT study protocol of an online adaptation of the intervention. The online version follows the same 9-session protocol, and the study provides illustrative examples of digital adaptations of the exercises in the toolkit.

## CBS approaches in cancer patients

Jiang et al. provided a meta-analysis of the efficacy of ACT in reducing psychological distress in adult patients with cancer. The study meta-analyzed 16 RCTs, conducted in six countries, in samples with various cancer types, with duration varying from 3 to 12 weeks, delivered in different formats, and showed significant reductions in anxiety and depression that were maintained at follow-up. The study concluded a significant increase in psychological flexibility; however, it should be noted that these studies used the AAQ-II measure, which has raised validity concerns (e.g., Cherry et al., 2021).

Bourgognon et al. expanded these results by conducting a mixed-method single-arm test of an ACT-informed process-based intervention with *N* = 40 French participants with mixed cancers. This was a low-intensity, 3-session, group intervention that followed a circular modality, which provided a new approach to delivery as an alternative to the structured unidirectional format usually implemented. The intervention was feasible and improved participants' quality of life and functioning.

## ACT and patient-centered research: qualitative assessment of change in muscle disease

Edwards et al. conducted, in the United Kingdom, a qualitative study to examine patients' experiences of an ACT intervention to improve the quality of life associated with muscle disease. Through inductive thematic analysis, following semi-structured interviews, results showed that participants perceived that ACT assisted them in making changes in ways that were consistent with the PF (e.g., shifts in perspective, increases in self-awareness and acceptance, and changes in behavior toward meaningful action).

## Beyond physical symptoms: ACT buffers stigma and increases self-compassion in chronic pain

Anderson et al. presented their results from an ACT intervention study conducted in the United Kingdom with *N* = 431 adults with chronic pain. Post-intervention, participants showed a decrease in stigma, which was negatively correlated with an increase in self-compassion. When accounting for the effect of psychological flexibility, self-compassion was no longer a significant predictor of changes in pain-related outcomes and depression. The authors provided a CBS-informed reading of these results by suggesting that self-compassion in chronic pain may be uniquely relevant only after reducing inflexibility processes and environmental factors.

## CBS in global crises: the case of the COVID-19 pandemic

Carvalho et al. provided evidence of 1-year longitudinal individual and contextual predictors of post-traumatic stress, anxiety and depression, and concluded that psychological flexibility and major life events, but not self-compassion, resilience or shame, predicted psychopathological symptoms (*N* = 61 Portuguese SARS-CoV-2 survivors). This tentatively suggests the importance of providing contextual measures not limited to self-regulation and coping, but also governmental-level measures to mitigate the impact of societal burden during global crises (e.g., parental burnout).
